# Economic evaluations of scaling up strategies of evidence-based health interventions: a systematic review

**DOI:** 10.1186/s12913-025-13024-w

**Published:** 2025-07-01

**Authors:** Adissa Bankolé, Blanchard Conombo, France Légaré, Maude Laberge

**Affiliations:** 1 Département de médecine sociale et préventive, Faculté de Médecine, Université Laval, Québec, Canada; 2 VITAM Centre de recherche en santé durable - Université Laval, Québec, Québec Canada; 3 Centre de recherche du CHU de Québec - Université Laval, Québec, Canada; 4 Département de médecine familiale et de médecine d’urgence, Université Laval, Québec, Canada

**Keywords:** Scaling, Cost-effectiveness, Evidence-based, Economic evaluation

## Abstract

**Background:**

Scaling seeks to extend the benefits of evidence-based interventions (EBIs) to larger populations, and often with the hope of achieving economies of scale. However, little is known about scaling costs. Our goal was to find scaling studies that focused on economic evaluations of scaling, their characteristics and the methods they applied.

**Methods:**

We performed a systematic review informed by the Joanna Briggs Institute and PRISMA reporting guidelines. We included all studies that conducted a full or partial economic evaluation of scaling an EBI in healthcare, applicable to any individual or organization in any country and setting. We included all study designs and imposed no restrictions on language. We conducted searches in Medline, Web of Science, Embase, Cochrane Library Database, PEDE, EconLIT, INHATA from their inception until November 12, 2024, including grey literature. Pairs of independent reviewers identified eligible studies and extracted data on study characteristics, scaling strategies, characteristics of economic evaluations and methods used. The methodological quality of included studies was evaluated using the British Medical Journal Checklist. Results were summarized using narrative synthesis.

**Results:**

Of 8,936 unique citations, thirteen studies meet our inclusion criteria: ten cost-effectiveness and three cost-analysis studies. Studies were performed in lower- or middle-income countries (LMIC) as well as in high-income countries and covered EBIs for infectious diseases, mental health, and colorectal cancer. All reported direct costs (e.g., health professional training costs) and indirect costs (e.g., capital costs) associated with scaling strategies. Four studies were of high quality, eight of moderate quality and one of poor quality.

**Conclusion:**

With the increased interest in scaling EBIs in health, there is an urgent need for more evaluations of costs associated with scaling, both in LMIC and in high-income countries, and a need for rigour in how these evaluations are performed.

**Supplementary Information:**

The online version contains supplementary material available at 10.1186/s12913-025-13024-w.

## Contributions to the literature

- ✓ In-depth study of existing Literature: The review provides a detailed analysis of the characteristics and methods in economic evaluations of scaling-up strategies.
- ✓ Identification of key elements specific to conducting an economic evaluation of scaling-up strategies of evidence-based interventions in health.
- ✓ Basis for Decision-making: By offering a clear understanding of cost components, the review can support evidence-informed health policies.

## Introduction

The World Health Organization recommends the adoption of an evidence-based approach for health promotion policies and practices [1]. Recent literature suggests that efforts to bridge the gap between knowledge and practice have produced a vast body of research on the efficacy and effectiveness of health interventions and their implementation in practice [2–4]. These efforts have produced a wide set of well-documented evidence-based interventions (EBIs) [2, 3, 5, 6]. However, health decision-makers are still not systematically implementing such evidence to benefit more people on a wider scale [2, 3, 5–9]. One way to fill this gap is to develop and implement strategies to scale evidence-based interventions (EBIs) [8, 10]. 

Scaling can focus on health or social outcomes such as health system functioning, access to medicines for tuberculosis infections, COVID-19 screening, or treatment of depression. It can also focus on food security, strengthening vulnerable communities, poverty, education and human development [11]. Recent studies have expanded this scope to include areas such as nationwide colorectal cancer screening programs and scaling of psychosocial interventions for mental health during perinatal periods, demonstrating the potential impact of EBIs in diverse domains and contexts​.

Many countries around the world have adopted the quintuple aim, which refocuses health interventions on health outcomes, patient experience, equity, the wellbeing of health providers, and efficiency, including cost-effectiveness. As part of the “efficiency” aim, cost is an aspect of implementation that is often left out of studies [12]. 

Scaling may involve expanding the geographic supply of an intervention and increasing the demand among the population for an intervention. There are two main approaches to scaling up health interventions: vertical and horizontal [12]. Scaling up using a vertical approach involves the simultaneous introduction of interventions throughout the system, resulting in the institutionalization of changes through policy, regulatory, financial, or health system changes while scaling up using a horizontal approach involves introducing an intervention in stages to different sites and groups [13]. 

In both cases, increasing the scope or scale of services to serve more individuals entails increasing costs [14]. Health systems face continuous strains and limited resource availability, and economic evaluations play an important role in informing health decision-makers about the trade-offs in costs and health benefits in selecting the EBI to be scaled up. A scaling up strategy refers to any process to extend the utilization, delivery or coverage of an evidence-based practice to other settings [15]. In addition, there is often the hope that scaling can produce economies of scale [16]. Thus, a rigorous evaluation of costs and cost-effectiveness is essential to the successful scale up of EBIs [1, 9, 17–20]. 

There are two primary categories of scaling-up costs: direct costs and indirect costs. Direct costs encompass both direct medical and direct non-medical costs, while indirect costs include a range of expenditures such as capital costs, utility costs, opportunity costs, maintenance costs, time costs, renovation costs, productivity costs and support personnel costs. Opportunity costs consist of costs related to missed opportunities related to choosing an alternative over other options. Productivity costs are those related to loss in the productivity. The distribution and classification of these costs depend on the nature of the intervention and the context in which it is implemented, necessitating a comprehensive understanding of cost structures to inform strategic decision-making [14]. These costs are specific to both the type of intervention and its setting.

Frequently, economic analysts must depend on assumptions that may not fully capture the intricacies of implementing evidence-based interventions (EBIs) on a large scale, due to the absence of comprehensive cost data for scaling up or the utilization of inadequate models [14, 21–27]. For instance, economic evaluations of strategies to scale up may suggest that the costs of scaling are a fixed proportion of the overall intervention costs [14, 27, 28]. In practice, strategies for scaling up may incur extra costs beyond those of the intervention itself, and these costs can differ significantly between different interventions and environments, potentially resulting in economies or diseconomies of scale [26]. Estimates of cost and cost-effectiveness can vary based on factors such as the type of evidence-based intervention (EBI) being scaled up, the size of the population targeted, disease prevalence or incidence, the effectiveness of the intervention, the geographical location, and the financial resources available and needed [9, 14, 15, 19, 26, 29–31]. Costs and estimates concerning infrastructure and human resources can differ depending on the operationalization and management of scaling up strategies. For instance, the expenses related to service delivery might fluctuate with changes in uptake, as well as the opportunity costs for providers and patients involved in the activities [9, 14, 15, 19, 26, 29–31]. Ultimately, theoretical frameworks for implementation and scale-up—which support thinking about and interpretating ‘real world’ complex data—consider economic constructs in scaling up strategies in different ways. For example, some frameworks consider cost (or resource) mobilization as a primary goal [12, 18], while others consider cost as an implementation outcome [32]. Frameworks also differ in how they evaluate potential benefits or effectiveness in relation to cost. Some focus on cost-benefit analysis (CBA), which considers a broad range of benefits beyond health outcomes, while others emphasize cost-effectiveness analysis (CEA), which typically measures health gains in terms of cost per QALY or LY gained [33, 34]. This variation leads to a broad diversity in the studies and methodologies used in the economic evaluations of scaling up strategies.

Thus, little is known about the economic aspects of the science and practice of scaling and very few studies on scaling consider costs [11]. More specifically, little is known about what such economic evaluations should include, as the cost-effectiveness of EBIs does not necessarily reflect the cost-effectiveness of the scaling up effort [9, 14, 15, 19, 22, 29, 30, 35]. Evidence from recent studies highlights this gap, urging the need for more nuanced and context-specific evaluations to inform effective and efficient scaling strategies​ [33, 36, 37]. 

Thus, we sought to identify the characteristics and methods applied in economic evaluations of scaling in healthcare. More specifically, this review aims to:

1. Identify and describe the economic evaluation methods used to assess scaling up strategies of EBIs in health;
2. Identify and describe the costs and cost components used in such economic evaluations;
3. Identify and describe environmental factors accounted for in such economic evaluations;
4. Discuss the strengths and limitations of each approach and explain reasons for variation in the reporting of economic evaluations of scaling up strategies for EBIs in health.

In this study, we applied an economic evaluation framework for scaling-up strategies, integrating cost-effectiveness (CEA) and cost-benefit analysis (CBA) to assess feasibility and impact. Our approach considers economic models, cost-effectiveness perspectives, and comprehensive cost assessments.

## Methods

### Study design

This systematic review adhered to the methodology recommended by the Joanna Briggs Institute [38, 39]. The study was registered in the Open Science Framework database (osf.io/fsq84) and the protocol has been published [40]. This review is reported following the guidelines of the Preferred Reporting Items for Systematic Reviews and Meta-Analyses (PRISMA) [41, 42]. We used PRISMA systematic review as we restricted the review to evaluating the effects of interventions, viz. economic evaluations. However, not all selected studies were full economic evaluations as 5 were only partial evaluations (cost analyses).

### Inclusion and exclusion criteria

Studies included in the review adhere to the eligibility criteria as described in Table 1.

**Table 1:** Eligibility criteria

	Inclusion criteria	Exclusion criteria
Population	Any individual, organisation, or system (directly or indirectly) involved in the delivery or receipt of any health services that was the target of the scale-up	NA
Intervention	Studies which evaluated scaling up strategies for an EBI	No restrictions on the type of EBI
Comparator	Economic evaluation of current practice or of alternative scaling up strategies	No restrictions on the type of comparator
Outcome	Incremental cost-effectiveness ratio, Incremental cost-utility ratio, net benefit, cost-benefit ratio, cost of illness averted, cost per quality-adjusted life year gained, cost per disability-adjusted life year averted.	Studies in which only the scaling up strategy’s effectiveness, adoption or health gain was reported
Study design	Partial economic evaluation designs, such as cost-minimization analysis, cost-comparison/cost-analysis, cost-outcome descriptions, cost-descriptions, budget impact analysis and full economic evaluations studies: CEA, CUA or CBA	Reviews, systematic reviews, qualitative studies, clinical effectiveness studies, critical reviews, editorials, commentaries, abstracts, protocols, and academic theses
Context	No restrictions	NA
Language	English language	NA

*EBI* Evidence-based intervention, *CEA* cost-effectiveness analysis, *CUA* cost-utility analysis, *CBA* cost-benefit Analysis, *NA* not applicable

### Search strategy

A comprehensive search was performed by an information specialist [40] in the Cochrane library, Medline, Embase, PEDE, EconLit and INHATA for studies published until November 12, 2024. Details of the search strategy are available in the published protocol [40]. A hand search was also performed to identify additional relevant articles from citations, bibliographies of included primary studies and other related literature. We developed a rigorous search strategy using a combination of the following two concepts: (1) scaling and (2) basic terms relating to economic evaluation. The search was limited to publications in English. The following sources were used in selecting the search terms: (1) reviews about scaling up [8, 21] and economic evaluations of scaling up; [21, 22, 43] (2) the scaling up expertise of our multidisciplinary team and (3) the thesaurus of the consulted bibliographic databases. All words and expressions proposed were tested and evaluated by an information specialist who collaborates with our team and is familiar with the key concepts of this systematic review before being accepted or rejected for the search strategy. We then developed a search strategy using an iterative consultation process with all co-authors until a final version was agreed upon. We decided to use the term “scaling” to designate all expressions referring to the spread of an innovation. It captures a wide span of expressions such as ‘scaling up,’ ‘scaling up strategy’, ‘scale up,’ ‘scale out,’ and ‘spread of technologies,’ as well as variations such as ‘widespread adoption of the technology’ or ‘rolling out the model of care.’ The concept of economic evaluation integrated all synonyms such as ‘cost evaluation,’ ‘economic analysis’ and ‘net benefit.’ Results of the search strategy are presented in the published protocol [40]. 

### Data management and study selection

Citations were managed using EndNote software (version X7.0.1, New York City: Thomson Reuters, 2011). Duplicates were identified and removed using electronic and manual screening. Unique records were then exported into Covidence (internet-based screening and data extraction tool).

All titles, abstracts, and full texts were reviewed by at least two independent reviewers (BC, AB, and ML) to identify eligible studies. Prior to selection, we developed and tested a pilot screening form against the eligibility criteria on a 7.5% random sample of the retrieved citations (title and abstracts) to validate the article inclusion process and ensure that reviewers shared a common understanding of the eligibility criteria. Then, reviewers independently screened the titles and abstracts using Covidence. Studies not fulfilling the eligibility criteria were excluded, and the full texts of the remaining studies were retrieved for further assessment. Articles with abstracts that did not appear to meet the criteria for exclusion, were ambiguous or whose abstract was missing were retained and reviewed in full. The full texts of retained studies were independently assessed for exclusion against inclusion/exclusion criteria by both reviewers. If information on eligibility was unavailable or unclear, study authors were contacted for additional information. Discrepancies between reviewers were resolved by consensus and the lead researcher (ML) adjudicated when necessary. Any reasons for exclusion were recorded in Covidence at the full-text stage.

### Data collection

A standardized data extraction template was developed with a detailed instruction manual and piloted in duplicate by the reviewers. This template, as described in the published protocol [40] was designed to ensure consistency in data collection. The extraction form was informed by the study objectives, eligibility criteria and the JBI-ACTUARI tool (Joanna Briggs Institute-Analysis of Cost, Technology and Utilisation Assessment and Review Instrument) [38]. Using this template, for each study, we extracted information on the key characteristics, the cost components included, the results for the outcomes of interest and the author’s conclusions [44]. The template was tested on a 10% random sample of the studies included for data collection. This pilot test helped us identify extraction items that were either missing from the template or likely to be confusing or unnecessary. Authors’ consensus was required before the template was modified. Any discrepancies between reviewers were resolved by consensus and the lead researcher adjudicated if necessary (ML).

### Data analyses

We employed structured narratives, statistics and tables to describe and summarize the characteristics of the studies included, as well as the key elements deemed crucial in methodologies for economic evaluations in healthcare [45]. Narrative synthesis was used to summarize the methods, emphasizing significant characteristics of the studies when relevant, focusing on differences and similarities, methodological weaknesses and, where possible, identifying the main drivers of cost-effectiveness outcomes. A meta-analysis was not performed due to the variability in cost estimates across and within different settings [46]. 

### Methodological quality of included studies

Two reviewers with methodological and content expertise (BC, AB) independently assessed the methodological quality of included studies using the 35-item British Medical Journal (BMJ) checklist which is the most used tool to assess the quality of economic evaluation studies [47]. Also known as the Drummond and Jefferson checklist, it is designed for full and partial economic evaluations as well as reports and commentaries on economic evaluations, and is thus aligned with our broad inclusion criteria [48]. The BMJ checklist items are based on consensus among international experts in health economics. It assigns a single point for methodological quality on each of the 35 items (range 0–35 points) organized in three sections: study design, data collection and analysis, and interpretation of results. Studies scoring over 26 (> 75%) were classified as high quality, studies scoring between 17 and 26 (50–75%) were classified as moderate quality and those with scores below 17 (˂50%) were classified as poor quality. These rating classifications are also reported in the literature on other quality appraisal checklists [49]. Of note, if available information was insufficient for a given item, no point was awarded for that item. Any disagreement in judgement between reviewers was resolved by consensus and the lead researcher adjudicated if necessary (ML).

## Results

### Results of the search

**Fig. 1:**
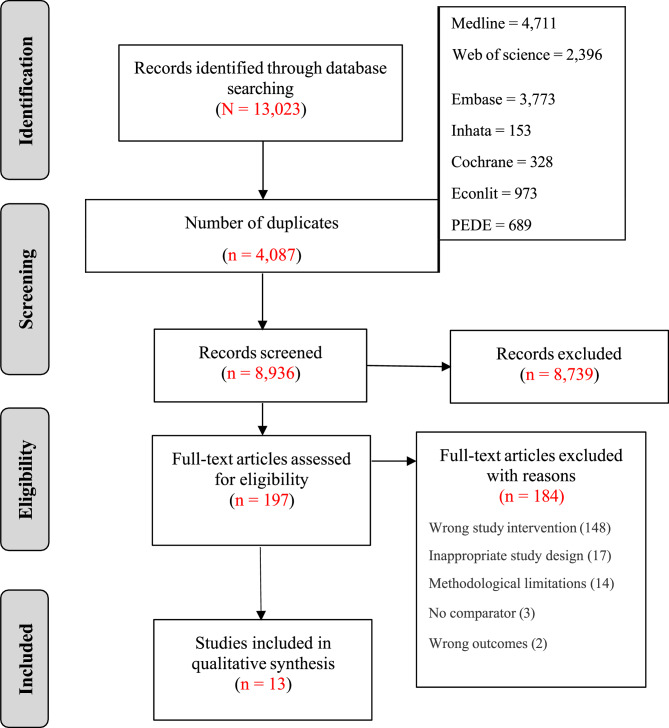
Preferred reporting items for systematic reviews and meta-analysis flow diagram

We identified 13,023 potentially relevant citations in our literature search and 8,936 unique citations after removing duplicates (Fig. 1). Of these, 197 (1.51%) were retained for full text review and 13 were considered eligible and included in the study.

### Characteristics of included studies

Thirteen economic evaluation studies were included in the qualitative synthesis (Table 2). Six studies [37, 50–54] defined scaling-up strategies, while in seven studies [33, 36, 55–59] the strategies were not clearly defined. We identified seven (54%) cost-effectiveness/cost utility analyses [36, 51–55, 58] four (31%) cost-analysis studies(partial economic evaluations) [37, 50, 56, 57] and two (15%) studies using simulation methodologies [33, 59]. Ten studies (77%) were conducted in public health settings​ [36, 37, 50–54, 56–58]. Three (23%) studies were conducted in South Africa [51–53] while others involved multiple countries, such as a study on COVID-19 self-testing across Brazil, Georgia, Malaysia, Ethiopia and the Philippines [58] and an analysis of hypertension treatment in 24 low-and middle-income countries [37]. Additional studies were conducted in individual countries, including China [36, 57], the United States [59], Bangladesh [52], Tanzania [53], England [50, 55] and Australia [56]).

**Table 2:** Results of economic evaluations of scaling up strategies

Author, year	Country	Type of EE	Population	Setting	Time horizon	Type of scaling	Scope of scaling	Intervention being scaled	Comparator	Incremental costs and outcomes	Authors’ conclusion
Alistar, 2014 [51]	South Africa	CEA; CUA	South Africa’s population aged 15 to 49 years old	Public health	20 years	Vertical	National	ART and PrEP access to all identified HIV-infected individuals (universal)	Access for those with CD4 counts > 350 cells/µL (guidelines)	$310 to $320 per QALY gained versus the status quo	Expanded ART coverage to individuals in early disease stages may be more cost-effective than current guidelines recommend.
Bauer, 2024 [55]	England	CEA	Women in perinatal period	Public health (NHS)	10 years	Horizontal	National	Midwives and health visitors delivering mental health interventions	Current government plans	Scaling up could reduce costs in the long run while improving maternal mental health outcomes	Encourages policy shifts to integrate mental health services into maternal care
Enns, 2024 [59]	USA	CEA	MSM	Urban jurisdictions	20 years (2023–2042)	Horizontal	Urban (Atlanta, Los Angeles, Miami)	HIV testing and PrEP adherence interventions	No additional efforts to mitigate implementation barriers	Cost-effective in some regions, with some interventions being cost-saving	Highlights the importance of optimizing HIV testing and PrEP implementation
Glaubius, 2016 [54]	South Africa	CEA	All individual aged 15–54 years in KwaZulu-Natal, South Africa	Public health	NR	Vertical	National	RPV PrEP scale-up for combination HIV prevention compared to prevention without PreP	Reference scenario without PrEP	Combined scale-up of conservative age-prioritized PrEP and second-line ART was very cost-effective ($1,169 per life-year gained) compared with second-line ART alone.	Scale-up of RPV PrEP in KwaZulu-Natal was very cost-effective among 20- to 29-year-old women, and cost-saving among individuals at highest behavioral risk.
Hansen, 2024 [58]	Brazil, Georgia, Malaysia, Ethiopia, Philippines	CA	General population requiring COVID-19 self-tests	public healthcare	One year for scaling scenarios	Horizontal	Municipal to national	Self-testing for COVID-19	Different distribution modalities	Cost per test ranged from $2.44 to $12.78, with potential cost savings under high-demand scenarios	Supports self-testing as a cost-effective strategy for scaling up COVID-19 diagnostics
Hutchinson 2024 [37]	24 LMICs	CA	Adults aged 30 + with hypertension	Public health	30 years	Horizontal	National	Strengthening hypertension care with different BP treatment thresholds (≥ 140, ≥ 150, ≥160 mmHg)	Current care standards	Scaling up to SBP ≥ 140 mmHg averted 2.6 million CVD events and 1.2 million deaths	Prioritizing severe hypertension treatment maximizes economic benefits in low-income countries
Knapp, 2024 [50]	England	CUA and CA	Family carers of people with dementia	Community-based dementia care	2015–2040	Horizontal	National	Scaling up the START intervention for dementia carers	Current level of carer support	Estimated to cost £9.4 M in 2020 but generate £68 M in annual savings with improved quality of life	Scaling up START reduces public sector costs and improves carers’ mental health
Lu, 2024 [33]	China	CEA	Population aged 45–74	Public healthcare	40 years (2020–2060)	Vertical	National	FIT and colonoscopy screening approaches	No screening	Colonoscopy achieved the highest CRC reduction but required the most resources; FIT was a cost-effective alternative	Supports large-scale CRC screening, with tailored implementation based on resource availability
Menon, 2014 [53]	Tanzania	CA	Males aged 10–49 in 8 Tanzanian regions	NR	15 years	Horizontal	National	Scaling-up VMMC for HIV prevention compared to current offer	Standard care comprising fixed sites in the existing public and private health delivery systems	Additional costs of en US$253.7 million for 2010 to 2015 and US$302.4 million for 2016 to 2025 to achieve targets	Given the health and economic benefits of investing in VMMC, the scale-up of services should continue to be a central component of the national HIV prevention strategy in Tanzania.
Nahar, 2017 [52]	Bang-ladesh	CA	NS (all individuals in rural areas in 2 districts)	Public health	18 months	Horizontal	Subnational	Behavior change communication intervention (TV broadcast) compared to other behavior change interventions.	No scaling-up intervention	To scale up a similar intervention in 30 districts where NiV spillover has occurred would cost between $2.6 and $3.5 million for one season. Placing the posters would cost $96,000 and only broadcasting the public service announcement through local channels in 30 districts would cost $26,000.	Broadcasting a TV public service announcement is a potential low-cost option to advance NiV prevention. It could be supplemented with posters and targeted interpersonal communication in districts with a high risk of NiV spillover.
Shih, 2023 [76]	Australia	CUA	PWID	Correctional facilities	40 years (2021–2060)	Horizontal	State (New South Whales)	DAA treatment and HCV testing in prison-based healthcare settings	Business-as-usual scenario maintaining HCV treatment at 2020 levels	AUD 12,968 per QALY gained	Scaling HCV testing and treatment in prisons is cost-effective and crucial for achieving national elimination goals
Zhang, 2024 [77]	China	CEA	HRH and MSM	Public healthcare	10 years (2019–2028)	Vertical	Municipal, six cities in eastern China	Enhance coverage to 99.90% for key HIV interventions (outreach, condoms, PrEP, ART, etc.)	Coverage remains at 2019 level	Incremental cost per HIV infection averted varies from 0.39 (MSM outreach in Shijiazhuang) to 53,976 (VCT in Foshan)	Cost-effectiveness of key interventions vary across cities and populations.
Zhao, 2024 [57]	Australia, Thailand, China	CEA	MSM	Public health	40 years	Vertical	National	Long-acting injectable PrEP vs. oral PrEP	Status quo of PrEP availability	Expanding oral PrEP use is cost-effective; injectable PrEP not currently affordable	Recommends expanding oral PrEP use, with country-specific HIV testing strategies

*ART* Antiretroviral therapy, *CA* Cost analysis, *CEA* Cost-effectiveness analysis, *CUA* Cost-utility analysis, *DAA* direct-acting antivirals, *EE* Economic evaluation, *HCV* Hepatitis C virus, *HRH* High-risk heterosexuals, *LMIC* Low- and Middle-Income Countries, *MMC* Medical Male Circumcision, *MSM* Men who have sex with men, *NHS* National Health Services, *NiV* Nipah virus infection, *NR* Not Reported, *PrEP* preexposure prophylaxis, *PWID* People who inject drugs, *RPV* rilpivirine, *VCT* Voluntary counseling and testing, *VMMC* Voluntary Medical Male Circumcision

Six studies (46%) assessed the costs of vertical scaling strategies at the national level [37, 50–53, 59] involving the simultaneous introduction of an intervention across an entire system, leading to institutional changes through policies, regulations, or financing. The remaining studies (54%) focused on horizontal or mixed scaling strategies, involving a phased introduction of an intervention across specific groups or sites​ [33, 36, 54–58]. Ten studies used simulation models [36, 37, 50–54, 57–59] to assess the costs of various alternatives. Three studies were implementation studies combined with economic evaluations [33, 55, 56]. These thirteen studies estimated the costs of scaling evidence-based interventions (EBIs), including antiretroviral therapy (ART) and pre-exposure prophylaxis (PrEP), voluntary medical male circumcision (VMMC), behavioral interventions to prevent Nipah virus (NiV), colorectal cancer screening programs, hypertension treatment, and psychosocial support interventions for caregivers of people with dementia. The interventions targeted diverse populations, including adults, adolescents, and specific at-risk groups (Table 2).

### Economic evaluations of scaling up strategies of EBIs in healthcare

Results of the incremental cost-effectiveness analyses suggest that scaling up these EBIs would generally be cost-effective. For instance, scaling access to ART for all identified HIV-infected individuals (aged 15 to 49) in early disease stages in South Africa was found to be more cost-effective than following current guidelines, which recommend access only for those with CD4 counts ≤ 350 cells/Μl, while PrEP could be cost-saving when delivered to individuals at increased risk of infection​ [51]. 

 Similarly, RPV PrEP for HIV prevention in KwaZulu-Natal (South Africa) demonstrated high cost-effectiveness compared to no PrEP and second-line ART scale-up, with an incremental cost-effectiveness ratio (ICER) of $1169 per life-year gained​ [ 52 ]. Glaubius et al. reported an ICER of $1,169 per LY gained [ 54 ] while Alistar et al. found a cost of $310–320 per QALY gained [ 51 ] both being considered as cost-effective. Given that a QALY generally exceeds a LY in value, the results of Alistar et al. suggest an even more favorable cost-effectiveness profile, warranting consistency in classification. To address this, we have refined the categorization accordingly. Additionally, both studies conducted sensitivity analyses to account for uncertainty in model parameters. Glaubius et al. included a multivariate sensitivity analysis demonstrating robustness under various assumptions [ 54 ] whereas Alistar et al. performed one-way sensitivity analyses but did not provide detailed results [ 51 ]. Future research should ensure systematic reporting of such analyses to enhance the reliability of cost-effectiveness estimates.

 Similarly, a behavior changes communication intervention, involving TV public service announcements to prevent Nipah virus transmission in Bangladesh, required an additional $26,000 and was considered a low-cost option​ [ 54 ]. Mass media campaigns, such as TV PSAs, are cost-effective demand-side interventions, whereas one-on-one education requires more resources, making scaling more expensive. Additionally, supply-side interventions, such as expanding healthcare infrastructure, involve higher upfront costs. Differentiating these strategies is key to optimizing scaling decisions.

 Cost analyses comparing scaling strategies highlight significant investments. A scaling-up campaign conducted in Tanzania to achieve 87.9% nationwide coverage of voluntary medical male circumcision (VMMC) compared to standard care would require an additional investment of $253.7 million between 2010 and 2015 and $302.4 million between 2016 and 2025 [ 53 ].

 Further analyses revealed the economic implications of scaling interventions in multiple countries. For COVID-19 self-testing, costs ranged from $5 to $10 per kit distributed, with workplace distribution being more cost-effective than distribution in schools or in clinics [ 58 ]. 

 Scaling hypertension treatment from an initial population of adults aged 30 years and older across 24 low- and middle-income countries showed initial costs ranging from $15 to $50 per patient, depending on the healthcare infrastructure and treatment cut-off points applied [ 37 ]. In England, scaling up psychosocial support for dementia caregivers involved initial costs of £4,500 per group-based program for 12 caregivers, with potential long-term savings of up to £10,000 per caregiver by delaying institutionalization and reducing healthcare utilization [ 33 ]. Colorectal cancer screening in China was estimated at $4 to $6 per FIT test and $50 to $100 per colonoscopy, with infrastructure costs reaching $50 million for nationwide coverage [ 59 ]. Meanwhile, scaling PrEP and HIV screening for men who have sex with men in Australia showed annual costs of $600 to $1,500 per individual depending on coverage. This cost estimation applies specifically to MSM at high risk of HIV infection, as described in the study’s methodology [ 57 ]. Eliminating hepatitis C in Australian prisons would require $10,000 per incremental patient treated, amounting to a total of $50 million for full national implementation​ [ 56 ]. 

### Costs and cost components reported

All included studies reported a variety of direct and indirect costs and cost components associated with the scaling up of EBIs. The types of costs (Table 3) to consider when scaling are adapted from the classification made by Johns et al., 2005 [14]. Direct healthcare costs including those related to program delivery, drugs, supplies, diagnosis, laboratory testing, resource allocation and health professional training were highlighted in several studies [33, 51–54]. Indirect costs, including infrastructure investments and logistical expenses, were emphasized in studies focusing on interventions implemented across multiple settings or countries​ [37, 52–54, 56, 58, 59]. For example, adaptations to existing systems and the expansion of services to underserved populations were recurring themes in the analysis of scaling strategies. Some studies also noted the significance of non-medical costs, such as those related to travel, accommodation, and stakeholder engagement, particularly in large-scale campaigns​ [52, 53]. Scaling up costs were generally higher in the short term due to the need for substantial initial investments. However, these costs often decreased over time as economies of scale were realized, especially in programs with high coverage or efficient targeting strategies​ [37, 52, 53, 57]. 

**Table 3:** Types of costs reported

Costs reported	Alistar (2014) [51]	Glaubius (2016) [54]	Menon (2014) [53]	Nahar (2017) [52]
Direct costs				
Direct medical costs				
Treatment costs	RS	RS	NR	NR
Costs of vaccine	RS	RS	NA	NA
Drugs costs	RS	RS	RS	NR
Diagnostic costs	RS	RS	NA	NR
Surgery costs	NA	RI	RS	NA
Laboratory testing costs	NR	NR	NA	NR
Vaccine wastage	NR	NR	NA	NR
Vaccine logistics	NR	NR	NA	NR
Vaccine delivery (including campaign)	NR	NR	NA	NR
Management of wastage	NR	NR	NR	NR
Other medical costs	RS	NR	NR	NR
Overhead costs	NR	RI	RS	NR
Direct non-medical costs				
Travel, meal and hotel	NR	NR	RS	R
Equipment and facilities	NR	RS	RS	R
Personnel costs	NR	RS	RS	R
Time loss	NR	NR	NR	NR
Staff costs	NR	NR	NR	R
Indirect costs				
Capital costs (equipment,buildings,land)	NR	RS	RS	R
Maintenance costs	NR	RS	RS	R
Utility costs	NR	RS	RS	R
Support personnel costs	NR	RS	RS	R
Management and supervision costs	NR	RS	RS	R
Renovating costs	NR	RS	RS	R
Time costs	NR	RS	RS	R
Opportuniy costs	NR	RS	NR	NR
Productivity costs	NR	NR	RS	R
Awareness costs (meetings, posters, announcement)	NR	NR	NR	R

Costs reported	Hutchinson (2024) [37]	Zhang (2024) [77]	Shih (2024) [56]	Knapp (2024) [50]
Direct costs				
Direct medical costs				
Treatment costs	RS	RS	RS	RS
Costs of vaccine	NA	NA	NA	NA
Drugs costs	RS	RS	RS	R
Diagnostic costs	R	RS	RS	R
Surgery costs	NA	NA	NA	NA
Laboratory testing costs	R	R	R	R
Vaccine wastage	NA	NA	NA	NA
Vaccine logistics	NA	NA	NA	NA
Vaccine delivery (including campaign)	NA	NA	NA	NA
Management of wastage	NA	NA	NA	NA
Other medical costs	R	RS	R	RS
Overhead costs	RS	RS	RS	RS
Direct non-medical costs				
Travel, meal and hotel	NR	NR	NR	NR
Equipment and facilities	R	RS	RS	RS
Personnel costs	RS	RS	RS	RS
Time loss	NR	NR	NR	NR
Staff costs	RS	RS	RS	RS
Indirect costs				
Capital costs (equipment,buildings,land)	RS	RS	RS	RS
Maintenance costs	R	RS	RS	RS
Utility costs	R	RS	RS	RS
Support personnel costs	RS	RS	RS	RS
Management and supervision costs	RS	RS	RS	RS
Renovating costs	NA	NA	NA	NA
Time costs	NA	NA	NA	NA
Opportuniy costs	RS	RS	RS	RS
Productivity costs	RS	RS	RS	RS
Awareness costs (meetings, posters, announcement)	RS	RS	RS	RS

Costs reported	Enns (2024) [59]	Lu (2024) [33]	Zhao (2024) [57]	Bauer (2024) [55]	Hansen (2024) [58]
Direct costs					
Direct medical costs					
Treatment costs	RS	NR	R	RS	R
Costs of vaccine	NA	NR	R	RS	R
Drugs costs	R	NR	R	R	R
Diagnostic costs	RS	R	R	RS	R
Surgery costs	NA	NA	NA	NA	NA
Laboratory testing costs	RS	R	R	RS	R
Vaccine wastage	NA	NA	NA	NA	NA
Vaccine logistics	NA	NA	NA	NA	NA
Vaccine delivery (including campaign)	NA	NA	NA	NA	NA
Management of wastage	NA	NA	NA	NA	NA
Other medical costs	R	NR	R	RS	R
Overhead costs	RS	R	RS	RS	R
Direct non-medical costs					
Travel, meal and hotel	NR	NR	NR	NR	NR
Equipment and facilities	R	R	RS	R	R
Personnel costs	RS	R	R	RS	RS
Time loss	NR	NR	NR	NR	NR
Staff costs	RS	R	RS	RS	R
Indirect costs					
Capital costs (equipment,buildings,land)	R	R	R	RS	R
Maintenance costs	RS	R	RS	R	R
Utility costs	R	R	R	R	R
Support personnel costs	RS	R	RS	R	RS
Management and supervision costs	RS	R	R	RS	RS
Renovating costs	NA	NA	NA	NA	NA
Time costs	NA	NA	NA	NA	NA
Opportuniy costs	RS	R	RS	R	R
Productivity costs	R	R	R	R	R
Awareness costs (meetings, posters, announcement)	RS	R	RS	RS	R

The results also showed that some combinations of strategies of scaling up are more attractive, cost-saving and offered significant benefit [33, 51, 54, 56]. 

### Environmental factors

Environmental factors include cultural, technological, demographic, and economic factors of the environment which could have an impact on the scaling up strategies. Several of the newly included studies acknowledged the influence of these factors. For instance, the success of COVID-19 self-testing distribution was influenced by the technological capacity and accessibility in different countries, as well as cultural acceptance of self-administered testing​ [58]. Similarly, scaling up hypertension treatment in low- and middle-income countries highlighted the role of demographic factors, such as age distribution and urban versus rural healthcare infrastructure, in determining the feasibility and cost-effectiveness of scaling the interventions​ [37]. The studies also showed how behavioral and social norms, such as rates of HIV testing and condom usage among men who have sex with men, affected the implementation and outcomes of targeted PrEP strategies in Australia​ [57]. Additionally, interventions addressing hepatitis C in prisons emphasized the importance of adapting strategies to specific demographic settings, such as high-risk incarcerated populations​ [56]. 

Although none of the studies explicitly modeled these environmental factors, their findings suggest that variations in disease prevalence, healthcare infrastructure, and societal attitudes significantly influence the design, scalability and cost-effectiveness of interventions. These results highlight the need for more explicit consideration of environmental factors in future economic evaluations of scaling-up strategies.

### Strengths/limitations of each approach

Eight cost-effectiveness studies were identified, conducted over periods ranging from 10 to 20 years (Table 3, Supplemental Digital Content). These studies [33, 37, 50, 51, 54, 55, 58, 59] used sensitivity analyses to address uncertainties in key parameters and presented results as incremental cost-effectiveness ratios. Most also performed calibrations to align their models with national-level disease prevalence and incidence data, ensuring robust projections. However, not all studies provided sufficient detail on the scaling-up strategies employed, limiting the ability to generalize findings.

Five cost-analysis studies focused primarily on quantifying the direct and indirect costs of implementing or scaling interventions [36, 52, 53, 56, 57]. These studies provided valuable insights into resource allocation and operational costs but lacked evaluations of the comparative effectiveness of alternative scale-up strategies.

While cost-effectiveness studies highlight trade-offs between costs and health outcomes, cost-analysis studies focus on feasibility and resource allocation. Together, they are complementary approaches, but the lack of standardization across methodologies remains a limitation.

### Variation in economic analysis methods

We observed variability in the economic analysis methods used in included studies (Table 4), highlighting the need for comprehensive evaluation frameworks.

Two studies employed vertical scaling approaches both with a 3% discount rate: one did not specify the perspective [51] while the other adopted a societal perspective.

The remaining studies focused on horizontal scaling, except Zhao et al. [57] which utilized both approaches. Among horizontal studies, perspectives varied: Menon et al. adopted a government perspective with no discount rate reported [53] while Hutchinson et al., Bauer et al., and Knapp et al. used a societal perspective with discount rates of 4%, 3.5%, and 3.5%, respectively [37, 50, 55]. Healthcare perspectives were adopted by Zhao et al., Lu et al., and Enns et al., with discount rates of 3%, 5%, and 3%, respectively [33, 57, 59]. Shih et al. applied a payer perspective with a 5% discount rate, and Hansen et al. used a provider perspective without applying a discount rate [58]. 

The variability in scaling approaches, perspectives, and discount rates highlights the need for standardized methods to ensure comparability and consistency in economic evaluations of scaling strategies.

**Table 4:** Methodological aspects of included studies

Studies	Type of economic analysis	Discount rate for effects	Discount rate for costs	Setting	Time horizon	Type of scaling-up	Scope of scaling-up	Study perspective	Clinical outcomes	Uncertainty analysis	Design
Alistar (2014) [51]	CEA; CUA	3%	3%	Public health	20 years	Vertical	National	NR	infections averted	Yes	Model-based
Glaubius (2016) [54]	Cost-effectiveness	3%	3%	Public health	NR	Vertical	National	Societal	Life-years gained	Yes	Model-based
Menon (2014) [53]	Cost-analysis	NR	NR	NR	15 years	Horizontal	National	Government	HIV infection averted	No	Unclear
Nahar (2017) [52]	Cost-analysis	NR	NR	Public health	18 months	Horizontal	Subnational	NR	NR	No	Trial-based
Enns (2024) [59]	CEA	3%	3%	Public health	20 years	Horizontal	Subnational	Societal	HIV infections averted	Yes	Model-based
Hansen (2024) [58]	Cost-analysis	NR	NR	Healthcare facilities & workplaces (COVID-19 self-testing)	1 Year	Horizontal	National	Government	COVID-19 cases detected	No	Micro-costing study
Lu (2024) [33]	CEA	3%	3%	Nationwide colorectal cancer screening program	40 years	Vertical	National	Healthcare system	Colorectal cancer cases averted	Yes	Microsimulation modeling
Zhao (2024) [57]	CUA	0–5%	0–5%	HIV prevention settings	40 years	Vertical	Subnational	Healthcare system	Life-years gained	Yes	Markov decision-analytic model
Bauer (2024) [55]	CEA	NR	NR	Maternal and child healthcare services	9 years	Horizontal	National	Government	Mental health improvements	Yes	Simulation modeling
Hutchinson (2024) [37]	CEA	3%	3%	Healthcare facilities for hypertension treatment	30 years	Vertical	National	Healthcare system	Cardiovascular events averted	Yes	Economic evaluation model
Zhang (2024) [77]	CEA	1,5%	1,5%	Urban healthcare settings	10 years	Horizontal	Subnational	Government	HIV infections prevented	Yes	Mathematical modeling
Shih (2024) [56]	CUA	5%, tested at 0%, 3%, 7%	5%, tested at 0%, 3%, 7%	Statewide prison healthcare system	40 years	Vertical	Subnational	Healthcare system	HCV cases averted	Yes	Dynamic modeling
Knapp (2024) [50]	CEA	3.5%	3.5%	Community-based dementia care	25 years	Horizontal	National	Governement	Carer mental health improvement	Yes	Trial-based economic evaluation

*CEA* Cost-effectiveness analysis, *CUA* Cost-effectiveness analysis

### Methodological quality of included studies

Of the studies included, four demonstrated high methodological quality, with scores exceeding 75% [33, 36, 56, 57]. Eight studies were classified as of moderate quality [37, 50, 51, 53–55, 58, 59] and one as of poor quality [52] according to the BMJ Checklist (Table 1, Supplemental Digital Content). The BMJ Checklist was chosen for its methodological rigor, alignment with the study type, comprehensive and standardized criteria, recognition in the scientific community, and ability to ensure transparency and comparability in the evaluation of studies.

All included studies clearly stated their research question in an answerable form and justified the perspective of their analysis. Six studies (46%) explicitly justified the choice of the model used and the key parameters on which they were based. However, several methodological shortcomings were identified: 62% of studies did not justify the type of economic evaluation chosen in relation to the research questions addressed, 69% did not discuss the economic importance of the research question, and 54% lacked details on the currency used for price adjustments, inflation, or currency conversion. Furthermore, 77% did not adequately discuss the choice of the discount rate, and 85% omitted details of statistical tests or confidence intervals for stochastic data.

## Discussion

We conducted a systematic review of the scientific literature to review the characteristics and methods applied in economic evaluations of scaling up strategies of EBIs in healthcare. We identified a total of thirteen full or partial economic evaluation studies. Most studies were conducted in LMICs and addressed a range of health issues, including infectious diseases, chronic conditions and public health interventions. Results showed a great variability in the quality of methodological reporting, and in the presentation of costs and cost components of scaling up strategies. These results lead us to make the following observations.

First, we found very few scaling studies that focused on economic evaluation, and those that did made little mention of scaling strategies. In some studies, scaling up strategies were not mentioned at all or else not clearly defined. In other studies, the aim was to increase the coverage of the intervention or program. “Scaling” is often interpreted as the same as “spread” in the literature. However, they present nuances in the methods and objectives that we considered in the selection of studies. These findings raise questions about the identification of EBIs and the scaling up strategies. In some cases, the scaling up is done in an emergency. For instance, in the COVID-19 pandemic context, various strategies were implemented to quickly scale up EBIs (such as vaccination campaigns or self-testing programs) but in the heist of the emergency, the strategies were not clearly planned, defined, and elaborated [58]. Moreover, our review found that some scaling-up strategies were explicitly focused on reaching specific high-risk populations, such as in the study by Glaubius et al. which targeted populations at highest risk of HIV infection, Similarly, the study by Menon et al. [53] highlighted the use of tailored approaches to scaling voluntary medical male circumcision to underserved regions. Last but not least, financing the scaling up of EBIs is important, and depends on the political will of governments [8, 9, 11, 47, 48]. Political will may mean expanding coverage of an EBI to another population [60]. Additionally, studies such as those by Bauer et al. and Knapp et al. emphasized the critical role of resource allocation and sustainability planning in ensuring the success of scaling up strategies, particularly in LMICs [50, 55]. 

Second, studies were predominantly from LMICs and focused on the prevention of infectious diseases. This is congruent with findings of a recent systematic review which also found the majority of scaling studies focused on infectious diseases in LMICs [11]. LMICs cannot afford multiple pilot studies of EBIs when resources are scarce, and outcomes are lethal. Thus, it would make sense that once a potentially beneficial EBI is identified to tackle a major public health threat such as HIV, that public health officials and policy makers would do as much as they can to scale this life-saving intervention [9]. The origins of the science and practice of scaling, including the rare economic evaluations of scaling, were born out of and driven by the urgent health needs in LMICs [9]. 

Expanding this expertise to high-income countries (HICs) could help address their specific challenges. While Canada and other HICs often serve as incubators for pilot studies, it is crucial that they also commit to scaling up effective EBIs to benefit larger populations. Expanding expertise from various countries offers insights into resource optimization and scalability, but requires adaptation to differences in financing, regulation, and health systems. Examples include task-shifting models for workforce efficiency and community-based interventions adapted for underserved populations in HICs, demonstrating the value of cross-learning with context-specific modifications [61]. 

Third, discrepancies in the quality of reporting were observed across the studies. While some showed moderate to high quality in areas such as design and data collection, many failed to justify their choice of economic evaluation methods or scaling strategies. Most focused primarily on the intervention itself, with limited attention to the broader considerations of scalability. These findings underscore the need for standardized reporting guidelines to ensure that both the intervention and its scalability are adequately addressed.

Although not within the scope of our review, Multi-Criteria Decision Analysis (MCDA) methods offer a complementary framework for evaluating health interventions by integrating multiple decision-making factors beyond cost-effectiveness. Studies have applied MCDA to assess scaling strategies, incorporating variables like budget impact, accessibility, broader health system effects [62, 63] and multiple dimensions of value, including patient-centered benefits, sustainability, and long-term feasibility [64].

To the best of our knowledge, this study represents the first attempt to identify the characteristics and methods applied in economic evaluations of the scaling up strategies of EBIs. Previous studies synthesized the costs and cost-effectiveness of increasing coverage of specific EBIs in healthcare [21, 22, 30, 65–67]. Most were conducted in LIMCs, and the results of these systematic reviews mirror our findings. Studies identified in these reviews focused mostly on national immunization programs [22, 65, 66], maternal, infant and children health programs [21] and HIV/AIDS prevention and care interventions [30, 67]. Like ours, these reviews revealed that economic evaluation studies vary greatly in their perspectives, scope, approaches, assumptions and cost categories, with their results often not presented in a way that allows easy comparison or generalized across settings and countries [21, 22, 30, 65–67]. However, in our review, the scope of EBIs expanded beyond infectious diseases to include interventions such as self-testing for COVID-19, mental health support programs, and non-communicable disease prevention. This broader inclusion reflects the growing recognition of the importance of scaling EBIs in various health contexts.

The studies identified in our review were conducted across diverse settings, including low- and middle-income countries as well as high-income countries such as England, China, and the United States. In LMICs, the focus remained predominantly on infectious diseases, reflecting the urgent need to scale up EBIs to address rapidly spreading health threats to their populations [8, 68–71]. In high-income countries, the studies explored a broader range of health issues, such as psychosocial interventions for perinatal mental health in England, colorectal cancer screening in China, and hepatitis C treatment in U.S. prisons. This expanded focus demonstrates the versatility of scaling strategies in addressing both chronic and infectious diseases. Moreover, given the financial constraints faced by LMICs scaling up existing EBIs remains a pragmatic and cost-effective strategy compared to investing in the development of new interventions [63]. In high-income countries, the emphasis on scaling strategies highlights the potential to optimize resource use and improve population health outcomes across various contexts. All our included studies reported direct (drug costs, supplies’ costs, diagnostic costs, costs of laboratory testing and health professional training costs) and indirect costs (capital costs, maintenance costs and utility costs) associated with scaling up strategies and discussed the methodological strengths and limitations of their studies. These results are consistent with those obtained by Johns et al. [14] who suggested that the major cost areas to consider when scaling EBIs are geography and infrastructure, fixed costs, human resources, and managing the process of scaling up.

We observed that scaling up costs were high in the early years. This can be explained by the higher costs of human resources, infrastructure, transportation, screening, costs of identifying and reaching specific populations [72, 73]. Previously, research funding was focused on pilot projects rather than scale-up. But this is changing as governments in high-income countries seek to reduce waste through economies of scale [11]. We noted that as programs become more routinized and high levels of scale-up are achieved, economies of scale may take effect bringing down average unit costs. Hence, there is a diminishing marginal cost in the scale up process. The higher the coverage scale, the lower the costs of scaling up [28]. It is also important to note that a costing study needs to assess the extent to which unused capacity in human resources would permit an intervention to be scaled up. Without trained health professionals and management personnel to implement a program, policy makers cannot realistically anticipate that a programme will be effectively scaled up [74]. Lack of human resources constitutes one of the most binding constraints to scaling up in the short run [74]. The cost of recruiting, training, and retaining skilled personnel must be accounted for when considering the cost of scaling [75]. 

### Strengths and limitations

We used a well-defined search strategy that encompassed multiple electronic databases to identify relevant economic evaluations. Furthermore, two independent reviewers applied the inclusion criteria and conducted a rigorous methodological quality assessment of included studies using the BMJ checklist that address internal and external validity aspects of full and partial economic evaluation studies [48]. Our search strategy also included peer-reviewed publications from various databases from their inception onwards.

Our review has a few limitations. We did not include some studies that focused on increasing coverage of health interventions because scaling up strategies or scaling up action plans were not clearly established or were not mentioned. Consequently, there is a possibility of having missed some potentially eligible studies. While several of the included studies addressed scaling strategies explicitly, many still lacked detailed descriptions of the stages of their scaling up processes. For instance, the distinction between the components being scaled up and the original interventions remained unclear in most cases.

This lack of clarity made it challenging to disaggregate our analysis in terms of the scope, pace, or extent of scaling up across the included studies. Instead, we focused on evaluating the methodological quality of the studies, which allowed us to identify notable strengths and weaknesses. Some newer studies demonstrated greater rigor in integrating economic evaluations with scaling strategies, yet inconsistencies in reporting persist. For example, variations in cost components and approaches to defining scaling remained a significant limitation.

Finally, considering the diversity of health contexts examined, this review remains limited in its capacity to provide comprehensive guidance on economic evaluations of scaling strategies. Nonetheless, we believe it is crucial to conduct such reviews to synthesize the economic methods used in scaling up strategies, showcase their diversity, and reflect on how future studies can improve the evaluation of these strategies. By doing so, we aim to lay the groundwork for a more standardized approach to economic evaluations of scaling up strategies in healthcare.

## Conclusion

Our study examined research that performed economic evaluations of scaling up strategies for EBIs in health and outlined the approaches used to estimate or assess scaling costs. Few studies on economic evaluations of scaling up strategies of EBIs were identified, encompassing a variety of health issues, including infectious diseases, chronic conditions, and mental health. These studies demonstrate the potential of economic evaluations to inform scalable interventions across different health contexts.

The findings of this study provide valuable insights to guide future research aimed at developing standardized tools for economic valuation that can be effectively integrated into scaling up frameworks and plans. Such tools are essential for supporting evidence-based decisions and optimizing the implementation of EBIs in healthcare systems.

## Supplementary Information

Supplementary Material 1.

## Data Availability

All data supporting the findings of this study are derived from previously published literature included in the systematic review. The full list of sources is provided in the manuscript and its supplementary materials. No new datasets were generated or analysed during the current study.

## References

[1] World Health Organization. Scaling up health services: Challenges and choices (Technical Brief No. 3). World Health Organization. 2008. https://apps.who.int/iris/handle/10665/43908.

[2] Massoud M, Donohue K, McCannon C. Options for Large-Scale spread of simple, high impact interventions. Bethesda: USAID Health Care Improvement Project; 2010.

[3] Eaton J, McCay L, Semrau M. Scale up of services for mental health in low-income and middle-income countries. Lancet. 2011;378:1592–603. 22008429 10.1016/S0140-6736(11)60891-X

[4] Greenhalgh T, Howick J, Maskrey N. Evidence based medicine: a movement in crisis? BMJ. 2014;348:g3725. 24927763 10.1136/bmj.g3725 PMC4056639

[5] Damschroder LJ, et al. Fostering implementation of health services research findings into practice: a consolidated framework for advancing implementation science. Implement Sci IS. 2009;4:50. 19664226 10.1186/1748-5908-4-50 PMC2736161

[6] Shaw J, Tepper J, Martin D. From pilot project to system solution: Innovation, spread and scale for health system leaders. BMJ Leader. 2018;2(2):87–90. 10.1136/leader-2017-000032.

[7] Whitworth J, Sewankambo NK, Snewin VA. Improving implementation: Building research capacity in maternal, neonatal, and child health in Africa. PLoS Med. 2010;7(7):e1000299. 10.1371/journal.pmed.1000299. 10.1371/journal.pmed.1000299 PMC2897765 20625547

[8] Ben Charif A, et al. Effective strategies for scaling up evidence-based practices in primary care: a systematic review. Implement Sci. 2017;12:139. 29166911 10.1186/s13012-017-0672-y PMC5700621

[9] Zomahoun HTV, Ben Charif A, Freitas A. The pitfalls of scaling up evidence-based interventions in health. Glob Health Action. 2019;12(1):1670449. 10.1080/16549716.2019.1670449. 10.1080/16549716.2019.1670449 PMC6781190 31575331

[10] Kruk ME, Yamey G, Angell SY. Transforming global health by improving the science of scale-up. PLoS Biol. 2016;14(3):e1002360. 10.1371/journal.pbio.1002360. 10.1371/journal.pbio.1002360 PMC4775018 26934704

[11] CORÔA RDC, et al. Evidence on scaling in health and social care: an umbrella review. Milbank Q. 2023;101:881–921. 37186312 10.1111/1468-0009.12649 PMC10509507

[12] World Health Organization & ExpandNet. Nine steps for developing a scaling‑up strategy (ISBN 9789241500319). World Health Organization. 2010. https://www.who.int/publications/i/item/9789241500319.

[13] World Health Organization, ExpandNet. Practical guidance for scaling up health service innovations. World Health Organization. 2009. https://apps.who.int/iris/handle/10665/44180.

[14] Johns B, Torres TT. Costs of scaling up health interventions: a systematic review. Health Policy Plan. 2005;20:1–13. 15689425 10.1093/heapol/czi001

[15] Roberts SLE, Healey A, Sevdalis N. Use of health economic evaluation in the implementation and improvement science fields—a systematic literature review. Implement Sci. 2019;14(1):72. 31307489 10.1186/s13012-019-0901-7 PMC6631608

[16] Freeman M, Savva N, Scholtes S. Economies of scale and scope in hospitals: an empirical study of volume spillovers. Manag Sci. 2021;67:673–97.

[17] Mangham LJ, Hanson K. Scaling up in international health: what are the key issues? Health Policy Plan. 2010;25:85–96. 20071454 10.1093/heapol/czp066

[18] Simmons R, Fajans P, Ghiron L. Scaling up health service delivery: from pilot innovations to policies and programmes. Geneva: World Health Organization; 2007.

[19] Victora CG, Hanson K, Bryce J, Vaughan JP. Achieving universal coverage with health interventions. Lancet. 2004;23–29(364):1541–8. 10.1016/S0140-6736(04)17279-6 15500901

[20] Milat AJ, Newson R, King L. Increasing the scale of population health interventions: A guide. Evidence CfEa. North Sydney: NSW Ministry of Health; 2014.

[21] Carroll G, et al. A systematic review of costing studies for implementing and scaling-up breastfeeding interventions: what do we know and what are the gaps? Health Policy Plan. 2020;35:461–501. 32073628 10.1093/heapol/czaa005

[22] Munk C, Portnoy A, Suharlim C. Systematic review of the costs and effectiveness of interventions to increase infant vaccination coverage in low- and middle-income countries. BMC Health Serv Res. 2019;19(1):741. 31640687 10.1186/s12913-019-4468-4 PMC6806517

[23] Ten Brink DC, et al. Cost-effectiveness and impact of pre-exposure prophylaxis to prevent HIV among men who have sex with men in asia: A modelling study. PLoS ONE. 2022;17:e0268240. 35617169 10.1371/journal.pone.0268240 PMC9135227

[24] Vassall A, Compernolle P. Estimating the resource needs of scaling-up HIV/AIDS and tuberculosis interventions in sub-Saharan africa: a systematic review for National policy makers and planners. Health Policy. 2006;79:1–15. 16388874 10.1016/j.healthpol.2005.11.005

[25] Marseille E, Larson B, Kazi DS, Kahn JG, Rosen S. Scaling up integrated prevention campaigns for global health: Costs and cost-effectiveness in 70 countries. BMJ Open. 2014;4(6):e003987. 10.1136/bmjopen-2013-003987. 10.1136/bmjopen-2013-003987 PMC4078786 24969782

[26] Turner HC, Toor J, Hollingsworth TD, Anderson RM. Economic evaluations of mass drug administration: the importance of economies of scale and scope. Clin Infect. 2018;66:1298–303. 10.1093/cid/cix1001 PMC5888956 29126255

[27] Turner HC, et al. Cost-effectiveness of scaling up mass drug administration for the control of soil-transmitted helminths: a comparison of cost function and constant costs analyses. Lancet Infect Dis. 2016;16:838–46. 26897109 10.1016/S1473-3099(15)00268-6

[28] Kumaranayake L. The economics of scaling up: cost Estimation for HIV/AIDS interventions. Aids. 2008;22(Suppl 1):S23–33. 18664950 10.1097/01.aids.0000327620.47103.1d

[29] Adam T, Ebener S, Johns B, Evans DB. Capacity utilization and the cost of primary care visits: Implications for the costs of scaling up health interventions. Cost Eff Resour Alloc. 2008;6(1):22. 19014524 10.1186/1478-7547-6-22 PMC2628647

[30] Salomon JA. Integrating economic evaluation and implementation science to advance the global HIV response. J Acquir Immune Defic Syndr. 2019;82(Suppl 3):S314–21. 31764269 10.1097/QAI.0000000000002219

[31] Hoomans T, Severens JL. Economic evaluation of implementation strategies in health care. Implement Sci. 2014;9(1):168. 25518730 10.1186/s13012-014-0168-y PMC4279808

[32] Proctor E, Silmere H, Raghavan R. Outcomes for implementation research: conceptual distinctions, measurement challenges, and research agenda. Adm Policy Ment Health. 2011;38:65–76. 20957426 10.1007/s10488-010-0319-7 PMC3068522

[33] Lu B, Luo J, Yan Y. Evaluation of long-term benefits and cost-effectiveness of nation-wide colorectal cancer screening strategies in China in 2020–2060: A modelling analysis. The Lancet Regional Health – Western Pacific. 2024;51:100853. 10.1016/j.lanwpc.2024.100853. 10.1016/j.lanwpc.2024.101172 PMC11380381 39247209

[34] Vicki B, Huong T, Miranda B, Rachel L, Marj M. A narrative review of economic constructs in commonly used implementation and scale-up theories, frameworks and models. Health Res Policy Syst. 2020;18(1):115. 32998752 10.1186/s12961-020-00633-6 PMC7528255

[35] Eisman AB, Kilbourne AM, Dopp AR, Saldana L, Eisenberg D. Economic evaluation in implementation science: making the business case for implementation strategies. Psychiatry Res. 2020;283. 10.1016/j.psychres.2019.06.008 PMC6898762 31202612

[36] Zhang Y, Wang L, Jiang Z. Exploration for the priority of HIV intervention: modelling health impact and Cost-Effectiveness — Six cities. China CDC Wkly. 2019;10. 10.46234/ccdcw2024.089 PMC11150166 38846361

[37] Hutchinson B, Walter A, Campbell N. Scaling hypertension treatment in 24 low-income and middle-income countries: economic evaluation of treatment decisions at three blood pressure cut-points. BMJ Open. 2024;14. 10.1136/bmjopen-2022-071036 PMC11029208 38626959

[38] Gomersall JS, et al. Conducting systematic reviews of economic evaluations. Int J Evid Based Heal. 2015;13:170–8. 10.1097/XEB.0000000000000063 26288063

[39] Moola S, Munn Z, Sears K. Conducting systematic reviews of association (etiology): the Joanna Briggs institute’s approach. Int J Evid Based Heal. 2015;13:163–9. 10.1097/XEB.0000000000000064 26262566

[40] Brundisini F, Giacomini M, Miller FA, Dodd S, Votruba N, Lavis JN. Economic evaluations of scaling up strategies of evidence-based health interventions: A systematic review protocol. BMJ Open. 2021;11(8):e047265. 10.1136/bmjopen-2020-047265. 10.1136/bmjopen-2021-050838 PMC8487175 34593499

[41] Liberati A, Altman DG, Tetzlaff J, Mulrow C, Gøtzsche PC, Ioannidis JPA, Clarke M, Devereaux PJ, Kleijnen J, Moher D. The PRISMA statement for reporting systematic reviews and meta-analyses of studies that evaluate health care interventions: Explanation and elaboration. PLoS Medicine. 2009;6(7):e1000100. 10.1371/journal.pmed.1000100. 10.1371/journal.pmed.1000100 PMC2707010 19621070

[42] Moher D, Liberati A, Tetzlaff J, Altman DG. Preferred reporting items for systematic reviews and meta-analyses: the PRISMA statement. Ann Intern Med. 2009;151:264. 19622511 10.7326/0003-4819-151-4-200908180-00135

[43] Glanville J, Fleetwood K, Yellowlees A. Development and testing of search filters to identify economic evaluations in MEDLINE and EMBASE. Ottawa, ON: Canadian Agency for Drugs and Technologies in Health; 2009.

[44] Gomersall JS, Jadotte YT, Xue Y, Lockwood S. The systematic review of economic evaluation evidence. In Joanna Briggs Institute Reviewers’ Manual. The Joanna Briggs Institute & The University of Adelaide. 2014. https://www.researchgate.net/publication/275655865_The_Systematic_Review_of_Economic_Evaluation_Evidence.

[45] Drummond M, Sculpher MJ, Claxton K, Stoddart GL, Torrance GW. Methods for the economic evaluation of health care programmes. Oxford: Oxford University Press; 2015.

[46] Higgins JPT, Green S. (Eds.). Cochrane handbook for systematic reviews of interventions (Version 5.1.0). The Cochrane Collaboration. Section 15.6.3: Meta-analysis of resource use and cost data. 2011. https://handbook-5-1.cochrane.org.

[47] Watts RD, Li IW. Use of checklists in reviews of health economic evaluations, 2010 to 2018. Value Health. 2019;22:377–82. 30832977 10.1016/j.jval.2018.10.006

[48] Drummond MF, Jefferson TO. Guidelines for authors and peer reviewers of economic submissions to the BMJ. The BMJ economic evaluation working party. Bmj. 1996;313:275–83. 8704542 10.1136/bmj.313.7052.275 PMC2351717

[49] Winser S, et al. Economic evaluations of physiotherapy interventions for neurological disorders: a systematic review. Disabil Rehabil. 2020;42:892–901. 30616401 10.1080/09638288.2018.1510993

[50] Knapp M, Lorenz-Dant K, Walbaum M. Scaling-up an evidence-based intervention for family carers of people with dementia: current and future costs and outcomes. Int J Geriatr Psychiatry. 2024;39. 10.1002/gps.6059 38279805

[51] Alistar SS et al. Comparative effectiveness and cost-effectiveness of antiretroviral therapy and pre-exposure prophylaxis for HIV prevention in South Africa. BMC Med. 2014;12. 10.1186/1741-7015-12-46 PMC4003813 24629217

[52] Nahar N, Asaduzzaman M, Sultana R. A large-scale behavior change intervention to prevent Nipah transmission in bangladesh: components and costs. BMC Res Notes. 2017;10. 10.1186/s13104-017-2549-1 PMC5485710 28651646

[53] Menon V, Gold E, Godbole R. Costs and impacts of scaling up voluntary medical male circumcision in Tanzania. PLoS ONE. 2014;9. 10.1371/journal.pone.0083925 PMC4011575 24802022

[54] Glaubius RL, Hood G, Penrose KJ. Cost-effectiveness of injectable preexposure prophylaxis for HIV prevention in South Africa. Clin Infect. 2016;63:539–47. 10.1093/cid/ciw321 PMC4967601 27193745

[55] Bauer A, Gregoire A, Tinelli M, Knapp M. Costs and benefits of scaling psychosocial interventions during the perinatal period in england: A simulation modelling study. Int J Nurs Stud. 2024;154. 10.1016/j.ijnurstu.2024.104733 38493516

[56] Shih STF, Stone J, Martin NK. Scale-up of Direct-Acting antiviral treatment in prisons is both Cost-effective and key to hepatitis C virus elimination. Open Forum Infect. 2024;11. 10.1093/ofid/ofad637 PMC10854215 38344130

[57] Zhao R, Fairley CK, Cook AR. Optimising HIV pre-exposure prophylaxis and testing strategies in men who have sex with men in australia, thailand, and china: a modelling study and cost-effectiveness analysis. Lancet Glob Health. 2024;12. 10.1016/S2214-109X(23)00536-3 38245115

[58] Hansen MA, Lekodeba NA, Chevalier JM. Cost of SARS-CoV-2 self-test distribution programmes by different modalities: a micro-costing study in five countries. Philipp BMJ Open. 2024;14. 10.1136/bmjopen-2023-078852 PMC11029185 38631825

[59] Enns B, Sui Y, Guerra-Alejos BC. Estimating the potential value of MSM-focused evidence-based implementation interventions in three Ending the HIV Epidemic jurisdictions in the United States: A model-based analysis. Journal of the International AIDS Society. 2024;27(Suppl 1):e26265. 10.1002/jia2.26265. 10.1002/jia2.26265 PMC11224592 38965982

[60] Fagan AA, Bumbarger BK, Barth RP. Scaling up Evidence-Based interventions in US public systems to prevent behavioral health problems: challenges and opportunities. Prev Sci. 2019;20:1147–68. 31444621 10.1007/s11121-019-01048-8 PMC6881430

[61] Begin, M. The political process: Monique Begin: A minister who knows how poor people feel. Canadian Medical Association Journal. 1978;118(11):1447–8. https://www.cmaj.ca/content/118/11/1447. PMC1818385 657040

[62] Wahlster P, et al. Balancing costs and benefits at different stages of medical innovation: a systematic review of Multi-criteria decision analysis (MCDA). BMC Health Serv Res. 2015;15:262. 26152122 10.1186/s12913-015-0930-0 PMC4495941

[63] Pinazo M-J, et al. Multi-criteria decision analysis approach for strategy scale-up with application to Chagas disease management in Bolivia. PLoS Negl Trop Dis. 2021;15:e0009249. 33770076 10.1371/journal.pntd.0009249 PMC8026069

[64] Rutten-van Mölken M, et al. Strengthening the evidence-base of integrated care for people with multi-morbidity in Europe using Multi-Criteria decision analysis (MCDA). BMC Health Serv Res. 2018;18:576. 30041653 10.1186/s12913-018-3367-4 PMC6057041

[65] Batt K, Fox-Rushby JA, Castillo-Riquelme M. The costs, effects and cost-effectiveness of strategies to increase coverage of routine immunizations in low- and middle-income countries: systematic review of the grey literature. Bull World Health Organ. 2004;82:689–96. 15628207 PMC2622984

[66] Pegurri E, Fox-Rushby JA, Damian W. The effects and costs of expanding the coverage of immunisation services in developing countries: a systematic literature review. Vaccine. 2005;23:1624–35. 15694515 10.1016/j.vaccine.2004.02.029

[67] Gomez GB, Borquez A, Case KK, Wheelock A, Vassall A, Hankins C. The cost and impact of scaling up pre-exposure prophylaxis for HIV prevention: A systematic review of cost-effectiveness modelling studies. PLOS Med. 2013;10(3):e1001401. 10.1371/journal.pmed.1001401. 10.1371/journal.pmed.1001401 PMC3595225 23554579

[68] Bitanihirwe BK. Scaling-up innovation as a means of tackling health inequalities. Commentary. Ann Ist super sanita. 2016;52:143–5. 27364384 10.4415/ANN_16_02_01

[69] Jarnheimer A, Kantor G, Bickler S, Farmer P, Hagander L. Frequency of surgery and hospital admissions for communicable diseases in a high- and middle-income setting. Br J Surg. 2015;102:1142–9. 26059635 10.1002/bjs.9845

[70] Milat AJ, Bauman A, Redman S. Narrative review of models and success factors for scaling up public health interventions. Implement Sci. 2015;10(1):113. 26264351 10.1186/s13012-015-0301-6 PMC4533941

[71] Nayagam S, Thursz M, Sicuri E. Requirements for global elimination of hepatitis B: a modelling study. Lancet Infect. 2016;16:1399–408. 10.1016/S1473-3099(16)30204-3 27638356

[72] Chisholm D, Lund C, Saxena S. Cost of scaling up mental healthcare in low- and middle-income countries. Br J Psychiatry. 2007;191:528–35. 18055957 10.1192/bjp.bp.107.038463

[73] Chola L, Pillay Y, Barron P, Tugendhaft A, Kerber K, Hofman K. Cost and impact of scaling up interventions to save lives of mothers and children: Taking South Africa closer to MDGs 4 and 5. Glob Health Action. 2015;8(1):27265. 10.3402/gha.v8.27265. 10.3402/gha.v8.27265 PMC4408314 25906769

[74] Hanson K, Ranson MK, Oliveira-Cruz V, Mills A. Expanding access to priority health interventions: a framework for Understanding the constraints to scaling-up. J Int Dev. 2003;15:1–14.

[75] Wilkinson D, Floyd K, Gilks CF. Antiretroviral drugs as a public health intervention for pregnant HIV-infected women in rural South africa: an issue of cost-effectiveness and capacity. Aids. 1998;12:1675–82. 9764788 10.1097/00002030-199813000-00016

[76] Shih STF, Stone J, Martin NK, Hajarizadeh B, Cunningham EB, Kwon JA, McGrath C, Grant L, Grebely J, Dore GJ, Lloyd AR, Vickerman P, Chambers GM. Scale-up of Direct-Acting Antiviral Treatment in Prisons Is Both Cost-effective and Key to Hepatitis C Virus Elimination. Open Forum Infect Dis. 2023;11(2):ofad637. 10.1093/ofid/ofad637. PMID: 38344130; PMCID: PMC10854215. 10.1093/ofid/ofad637 PMC10854215 38344130

[77] Zhang Y, Wang L, Jiang Z, Yan H, Liu X, Gu J, Wang G, Cheng X, Leng Q, Long Q, Liang Z, Wang J, Liang L, Qiu Y, Chen L, Hong H. Exploration for the Priority of HIV Intervention: Modelling Health Impact and Cost-Effectiveness - Six Cities, Eastern China, 2019-2028. China CDC Wkly. 2024;6(20):463-468. 10.46234/ccdcw2024.089. PMID: 38846361; PMCID: PMC11150166. 10.46234/ccdcw2024.089 PMC11150166 38846361

