# Supplementary material for: Economic evaluations of scaling up strategies of evidence-based health interventions: a systematic review
Source: BMC Health Serv Res. 2025 Jul 1;25:836. doi: 10.1186/s12913-025-13024-w (PMC12210681; doi:10.1186/s12913-025-13024-w)
Supplement: Supplementary file 1 — Supplementary Material 1. [file 12913_2025_13024_MOESM1_ESM.docx]

**Supplemental digital content**

**Table 1. BMJ Checklist - Quality appraisal of studies using the checklist of Drummond and Jefferson**

| **Item**  **Study** | **1** | **2** | **3** | **4** | **5** | **6** | **7** | **8** | **9** | **10** | **11** | **12** | **#13** | **14** | **15** | **16** | **17** | **18** | **19** | **20** | **21** | **22** | **23** | **24** | **25** | **26** | **27** | **28** | **29** | **30** | **31** | **32** | **33** | **34** | **35** | **Quality**  **Score^*^** |
| --- | --- | --- | --- | --- | --- | --- | --- | --- | --- | --- | --- | --- | --- | --- | --- | --- | --- | --- | --- | --- | --- | --- | --- | --- | --- | --- | --- | --- | --- | --- | --- | --- | --- | --- | --- | --- |
| Alistar 2014 | ✓ | ✓ | ✓ | ✓ | ✓ | ✓ | 🗶 | ✓ | NA | 🗶 | ✓ | ✓ | ✓ | NA | NA | 🗶 | 🗶 | 🗶 | 🗶 | 🗶 | 🗶 | ✓ | ✓ | 🗶 | NA | 🗶 | ✓ | ✓ | ✓ | ✓ | ✓ | ✓ | ✓ | ✓ | ✓ | 21/35 |
| Glaubius 2016 | ✓ | 🗶 | ✓ | ✓ | ✓ | ✓ | 🗶 | ✓ | NA | ✓ | ✓ | 🗶 | ✓ | 🗶 | ✓ | 🗶 | ✓ | ✓ | ✓ | 🗶 | ✓ | ✓ | ✓ | 🗶 | 🗶 | 🗶 | ✓ | ✓ | ✓ | ✓ | ✓ | ✓ | ✓ | ✓ | ✓ | 25/35 |
| Menon 2014 | ✓ | 🗶 | ✓ | 🗶 | ✓ | ✓ | ✓ | ✓ | ✓ | NA | ✓ | ✓ | ✓ | NA | NA | 🗶 | ✓ | 🗶 | 🗶 | ✓ | ✓ | ✓ | ✓ | 🗶 | NA | 🗶 | 🗶 | NA | NA | ✓ | ✓ | ✓ | ✓ | ✓ | 🗶 | 20/35 |
| Nahar 2017 | ✓ | 🗶 | ✓ | ✓ | ✓ | ✓ | 🗶 | NA | NA | NA | ✓ | NA | ✓ | NA | NA | ✓ | ✓ | ✓ | 🗶 | NA | NA | ✓ | 🗶 | 🗶 | 🗶 | 🗶 | 🗶 | 🗶 | 🗶 | ✓ | ✓ | ✓ | ✓ | ✓ | ✓ | 17/35 |
| Lu 2024 | ✓ | ✓ | ✓ | ✓ | ✓ | ✓ | ✓ | ✓ | ✓ | ✓ | ✓ | ✓ | ✓ | ✓ | 🗶 | NA | 🗶 | ✓ | ✓ | ✓ | ✓ | ✓ | ✓ | ✓ | ✓ | ✓ | NA | ✓ | ✓ | ✓ | ✓ | ✓ | ✓ | ✓ | ✓ | 26/35 |
| Zaho | ✓ | ✓ | ✓ | ✓ | ✓ | ✓ | ✓ | ✓ | ✓ | ✓ | NA | ✓ | ✓ | ✓ | 🗶 | NA | 🗶 | ✓ | ✓ | ✓ | ✓ | ✓ | ✓ | ✓ | ✓ | ✓ | NA | ✓ | ✓ | ✓ | ✓ | ✓ | ✓ | ✓ | ✓ | 30/35 |
| Bauer 2024 | ✓ | ✓ | ✓ | ✓ | ✓ | ✓ | ✓ | ✓ | ✓ | ✓ | ✓ | ✓ | ✓ | ✓ | * | NA | * | ✓ | ✓ | ✓ | ✓ | ✓ | ✓ | ✓ | ✓ | ✓ | NA | ✓ | ✓ | ✓ | ✓ | ✓ | ✓ | ✓ | ✓ | 29/35 |
| Zhang 2024 | ✓ | ✓ | ✓ | ✓ | ✓ | ✓ | ✓ | ✓ | ✓ | ✓ | ✓ | ✓ | ✓ | ✓ | * | ✓ | ✓ | ✓ | ✓ | ✓ | ✓ | ✓ | ✓ | ✓ | ✓ | ✓ | NA | ✓ | ✓ | ✓ | ✓ | ✓ | ✓ | ✓ | ✓ | 27/35 |
| Knapp 2024 | ✓ | ✓ | ✓ | ✓ | ✓ | ✓ | ✓ | ✓ | ✓ | ✓ | ✓ | ✓ | ✓ | ✓ | ✓ | NA | NA | ✓ | ✓ | ✓ | ✓ | ✓ | ✓ | ✓ | ✓ | ✓ | ✓ | ✓ | ✓ | ✓ | ✓ | ✓ | ✓ | ✓ | ✓ | 29/35 |
| Shil 2024 | ✓ | ✓ | ✓ | ✓ | ✓ | ✓ | ✓ | ✓ | ✓ | ✓ | ✓ | ✓ | ✓ | ✓ | 🗶 | NA | 🗶 | 🗶 | ✓ | ✓ | ✓ | ✓ | ✓ | ✓ | ✓ | ✓ | NA | ✓ | ✓ | ✓ | ✓ | ✓ | ✓ | ✓ | ✓ | 30/35 |
| Hansen 2024 | ✓ | ✓ | ✓ | ✓ | ✓ | ✓ | ✓ | ✓ | ✓ | ✓ | ✓ | 🗶 | ✓ | ✓ | 🗶 | NA | 🗶 | 🗶 | 🗶 | 🗶 | 🗶 | 🗶 | 🗶 | 🗶 | NA | NA | NA | ✓ | ✓ | ✓ | ✓ | ✓ | ✓ | ✓ | ✓ | 27/35 |
| Hutchinson 2024 | ✓ | ✓ | ✓ | ✓ | ✓ | ✓ | ✓ | ✓ | ✓ | ✓ | NA | ✓ | ✓ | ✓ | 🗶 | NA | 🗶 | ✓ | ✓ | ✓ | ✓ | ✓ | ✓ | ✓ | ✓ | ✓ | ✓ | ✓ | ✓ | ✓ | ✓ | ✓ | ✓ | ✓ | ✓ | 28/35 |

✓, Yes or Stated; 🗶, No or Not stated; NA, Not Appropriate; *, number of items clearly and well reported

Item 1. The research question is stated

Item 2. The economic importance of the research question is stated

Item 3. The viewpoint(s) of the analysis are clearly stated and justified

Item 3. The viewpoint(s) of the analysis are clearly stated and justified

Item 4. The rationale for choosing the alternative programmes or interventions compared is stated

Item 5. The alternatives being compared are clearly described

Item 6. The form of economic evaluation used is stated

Item 7. The choice of form of economic evaluation is justified in relation to the questions addressed

Item 8. The source(s) of effectiveness estimates used are stated

Item 9. Details of the design and results of effectiveness study are given (if based on a single study)

Item 10. Details of the method of synthesis or meta-analysis of estimates are given (if based on an overview of a number of effectiveness studies

Item 11. The primary outcome measure(s) for the economic evaluation are clearly stated

Item 12. Methods to value health states and other benefits are stated

Item 13. Details of the subjects from whom valuations were obtained are given

Item 14. Productivity changes (if included) are reported separately

Item 15. The relevance of productivity changes to the study question is discussed

Item 16. Quantities of resources are reported separately from their unit costs

Item 17. Methods for the estimation of quantities and unit costs are described

Item 18. Currency and price data are recorded

Item 19. Details of currency of price adjustments for inflation or currency conversion are given

Item 20. Details of any model used are given

Item 21. The choice of model used and the key parameters on which it is based are justified

Item 22. Time horizon of costs and benefits is stated

Item 23. The discount rate(s) is stated

Item 24. The choice of rate(s) is justified

Item 25. An explanation is given if costs or benefits are not discounted

Item 26. Details of statistical tests and confidence intervals are given for stochastic data

Item 27. The approach to sensitivity analysis is given

Item 28. The choice of variables for sensitivity analysis is justified

Item 29. The ranges over which the variables are varied are stated

Item 30. Relevant alternatives are compared

Item 31. Incremental analysis is reported

Item 32. Major outcomes are presented in a disaggregated as well as aggregated form

Item 33. The answer to the study question is given

Item 34. Conclusions follow from the data reported

Item 35. Conclusions are accompanied by the appropriate caveat
